# T Lymphocyte Inhibition by Tumor-Infiltrating Dendritic Cells Involves Ectonucleotidase CD39 but Not Arginase-1

**DOI:** 10.1155/2015/891236

**Published:** 2015-09-30

**Authors:** Malika Trad, Alexandrine Gautheron, Jennifer Fraszczak, Darya Alizadeh, Claire Larmonier, Collin J. LaCasse, Sara Centuori, Sylvain Audia, Maxime Samson, Marion Ciudad, Francis Bonnefoy, Stéphanie Lemaire-Ewing, Emmanuel Katsanis, Sylvain Perruche, Philippe Saas, Bernard Bonnotte

**Affiliations:** ^1^INSERM UMR1098, University of Bourgogne Franche-Comté, EFS Bourgogne Franche-Comté, 25020 Besançon, France; ^2^Department of Pediatrics, Steele Children's Research Center, Department of Immunobiology, BIO5 Institute and Arizona Cancer Center, University of Arizona, Tucson, AZ 85724, USA; ^3^INSERM UMR866, University of Bourgogne Franche-Comté, 21000 Dijon, France

## Abstract

T lymphocytes activated by dendritic cells (DC) which present tumor antigens play a key role in the antitumor immune response. However, in patients suffering from active cancer, DC are not efficient at initiating and supporting immune responses as they participate to T lymphocyte inhibition. DC in the tumor environment are functionally defective and exhibit a characteristic of immature phenotype, different to that of DC present in nonpathological conditions. The mechanistic bases underlying DC dysfunction in cancer responsible for the modulation of T-cell responses and tumor immune escape are still being investigated. Using two different mouse tumor models, we showed that tumor-infiltrating DC (TIDC) are constitutively immunosuppressive, exhibit a semimature phenotype, and impair responder T lymphocyte proliferation and activation by a mechanism involving CD39 ectoenzyme.

## 1. Introduction

Dendritic cells (DC) are professional antigen presenting cells (APC) specialized in the capture, processing, and presentation of antigens to specific T lymphocytes [1–3]. DC, therefore, orchestrate the T-cell fate through their activation, proliferation, and subset polarization resulting in competent adaptive responses. However, in many solid tumors, including breast and lung cancers, infiltrating DC (TIDC) exhibit an abnormal phenotype and impaired function [4–7]. The immunosuppressive tumor microenvironment can indeed alter the differentiation and activation of DC, which become unable to adequately license antitumoral T lymphocytes [8–13]. The most commonly observed defects of TIDC include an immature phenotype defined by the lack or reduced expression of costimulatory molecules (including CD80, CD86, and CD40), an impaired production of proinflammatory cytokines (such as IL-12), and an altered antigen-presenting machinery [7, 8, 14–22]. Numerous studies have also suggested that TIDC actively suppress immune responses by potentiating alternative immunosuppressive mechanisms, hereby contributing to tumor escape from immune surveillance [8, 18, 21, 23]. Previous reports have indicated that DC associated with human mammary carcinoma express indoleamine 2,3-dioxygenase (IDO) leading to tryptophan depletion, which subsequently results in T lymphocyte inhibition [24]. Despite exhibiting a mature phenotype, arginase-1-expressing TIDC, described in the NeuT mammary murine tumor model, can suppress T lymphocyte proliferation by depleting arginine from the environment. Alternatively, ovarian cancer-associated DC block T-cell proliferation by a programmed cell death-1- (PD-1-) dependent mechanism [25]. CD39 expression and the associated ATP hydrolysis and adenosine production, a potent anti-inflammatory molecule, have also been proposed to contribute to the mechanisms responsible for the suppressive activity of immune cells [26]. However, the expression of CD39 by tumor-associated DC and the implication of this enzyme in the tumor-promoting activity of TIDC are unclear. TIDC have also been involved in the generation of immunosuppressive regulatory T lymphocytes (Treg) capable of suppressing antitumor immunity and therefore promoting tumor development [27–29]. Overcoming TIDC-mediated immunosuppression is essential for the implementation of efficient immunobased anticancer interventions and requires a better understanding of the T-cell suppressive mechanisms employed by these cells.

We here present results indicating that, in the mouse lung LLC and mammary 4T1 cancer models, CD11c^+^ DC infiltrating tumors exhibit a semimature phenotype (intermediary expression of MHC-II, CD80, CD86, and CD83) and significantly suppress T lymphocyte activation* in vitro* by a mechanism involving CD39 ectoenzyme.

## 2. Materials and Methods

### 2.1. Mice

Female BALB/c and C57BL/6 mice were purchased from Charles River (Saint-Germain-sur-l'Arbresle, France) and housed in the University of Burgundy animal facility (Dijon, France). Animal use and handling were approved by the local veterinary committee and were performed according to the European laws for animal experimentation.

### 2.2. Cell Lines and Tumor Implantation

The mammary carcinoma (4T1) and Lewis Lung Cancer (LLC) cell lines were obtained from the ATCC (American Tissue Cell Culture) and cultured in RPMI 1640 (Lonza) supplemented with 10% FBS (Lonza) and 1x antibiotic-antimycotic (Gibco) (complete medium, CM) at 37°C, 5% CO_2_. Mice were inoculated with 1 × 10^6^ 4T1 (in both sides of the abdominal mammary gland) or with 1 × 10^6^ LLC (left and right flank) cells. After 2 weeks, tumors were harvested and processed. DC were isolated as outlined hereafter.

### 2.3. DC Isolation

Control DC were isolated from the spleen of tumor-free mice and TIDC were purified from 4T1 or LLC tumors. Tissues were collected, washed in sterile RPMI 1640 (Lonza), minced into small fragments, and incubated in a solution of type I collagenase (1.5 mg/mL) (Sigma-Aldrich) with continuous shaking (37°C, 45 min) in CM. The obtained single-cell suspension was filtered through a 100 *μ*m cell strainer (BD Biosciences) and cells were washed twice in CM. TIDC and splenic DC (spDC) were then purified based on CD11c expression using anti-CD11c magnetic microbeads (Miltenyi Biotec) and an autoMACS Separator following the manufacturer's instructions (Miltenyi Biotec).

### 2.4. Immunofluorescence

Tumors were dissected from euthanized mice and immediately embedded in tissue-Tek (O.C.T.; Sakura Finetek, Inc., Torrance, CA), snap-frozen in liquid nitrogen, and stored at −80°C. All samples were cut into 5 *μ*m thick sections. Immediately before staining, sections were fixed with cold methanol/acetone for 10 min. After washing in PBS, slides were incubated for 30 min with PBS supplemented with 3% BSA. Sections were then stained with anti-CD11c-biotin (clone HL3, BD Biosciences) (1 hour) followed by 45 minutes staining with streptavidin coupled with Alexa Fluor 568 and examined using a Zeiss Axiovert 200 inverted fluorescent microscope. Pictures were taken with an AxioCam HRml digital camera.

### 2.5. T-Cell Proliferation Assays

Splenocytes from naïve BALB/c or C57BL/6 mice were enriched for T lymphocytes using nylon wool columns. T cells, used as responders, were plated in CM at 1 × 10^5^ cells/well in 96-well round bottom plates with anti-CD3/CD28 T-cell expander beads (Invitrogen) and cultured for 4 days with or without DC. Cultures were pulsed with [^3^H]-Thymidine (1 *μ*Ci/well) for the last 12 hours. [^3^H]-Thymidine incorporation was measured using a liquid scintillation counter. Percentages of T-cell proliferation were calculated compared to [^3^H]-Thymidine incorporation in T cells cultured with anti-CD3/CD28 T-cell expander beads considered as 100%. In other experiments, total T lymphocytes were labeled with CellTrace Violet (CellTrace, Invitrogen) and cultured with DC. After 5 days, T-cell proliferation was detected by flow cytometry (LSRII flow cytometer, BD Biosciences) and analyzed using the ModFit software. Specific inhibitors were used at the following concentrations to suppress/neutralize various immunosuppressive modulators: polyoxometalate-1 (POM-1, CD39 inhibitor, 50 *μ*M, Sigma-Aldrich), adenosine 5′-(*α*,*β*-methylene)diphosphate (APCP, CD73 inhibitor, 10 *μ*M, Sigma-Aldrich, Saint Louis, USA), N^G^-methyl-l-arginine (NMMA, 500 *μ*M, nitric oxide synthase (NOS) inhibitor, Sigma-Aldrich), N(omega)-hydroxy-nor-L-arginine (nor-NOHA, arginase-1 inhibitor, 50 *μ*M, Calbiochem, San Diego, USA) and 1-methyl-tryptophan (1-MT, inhibitor of IDO, 200 *μ*M, Sigma-Aldrich).

### 2.6. Antibodies and Flow Cytometry Analysis

Cells (1 × 10^6^) were washed in PBS containing 0.5% BSA. To prevent nonspecific binding cells were incubated with 5% normal rat serum for 10 min at RT. Cells were then stained (45 min, on ice) with the appropriate fluorochrome-conjugated Ab (anti-CD11c-APC, anti-CD11b FITC, anti-CD86 PE, anti-PDCA-1 FITC, anti-Gr-1 PB, anti-MHC-II PE, anti-CD4 PB, anti-CD8 FITC, anti-CD25 PE, and anti-CD3 FITC Ab (eBioscience)). Cells were washed and analyzed using a LSRII cytometer (BD Biosciences). Data analysis was performed with the FlowJo software (version 5.7.2).

### 2.7. Cytokine Assays

The concentration of IL-12, IL-10, and IFN-*γ* in the culture supernatants was determined by enzyme-linked immunosorbent assay (ELISA) kits according to the manufacturers' procedures (eBiosciences).

### 2.8. ATP, ADP, and Adenosine Assays

The concentration of ATP and ADP in the TIDC and T-cell coculture supernatants were determined using a fluorometric assay kit (Abcam, Cambridge, UK) and adenosine concentration was evaluated using a chemiluminescence detection kit (DiscoveRx, Birmingham, UK) according to the manufacturer's procedures.

### 2.9. Western Blotting

Freshly isolated spDC, TIDC, or murine normal hepatocytes were lysed at 4°C for 20 min in a RIPA buffer containing protease inhibitors (2.5 *μ*g/mL pepstatin, 10 *μ*g/mL aprotinin, 5 *μ*g/mL leupeptin, and 0.1 mM PMSF). After centrifugation, protein concentration in the supernatant was determined using a Bio-Rad protein assay (Hercules). Thirty micrograms of proteins was separated by SDS-PAGE (12% polyacrylamide gel for IDO and arginase-1 detection and 7% for inducible NOS (iNOS) detection). Proteins were electrotransferred onto a nitrocellulose membrane. The membrane was blocked in Tris Buffered Saline (TBS) with 1% Tween-20 and 5% skim milk and incubated overnight (4°C) with anti-mouse-IDO monoclonal antibody (1 : 5000, Enzo Life Sciences), anti-arginase-1 (1 : 2000, R&D Systems), and anti-iNOS (1 : 500, R&D Systems). The membrane was washed and incubated (room temperature, 2 h) with HRP-conjugated secondary antibody. Immunoblots were then developed using an enhanced chemiluminescence (ECL) reagent kit from Santa Cruz Biotechnology, according to the manufacturer's protocol.

### 2.10. HPLC Measurement

Tryptophan, ornithine, and arginine were measured by high-pressure liquid chromatography (HPLC) as indicators of IDO and arginase-1 activity. After a 5-day TIDC and T-cell coculture, the supernatant was collected and tryptophan, ornithine, and arginine concentrations were measured. In brief, 200 *μ*L of cell supernatant was subjected to a deproteinization step using a 30% sulfosalicylic acid solution (Sigma). Then, supernatants were diluted with Jeol sampling buffer (JEOL) containing 0.2 *μ*mol/mL of aminoethyl cysteine and glucosaminic acid (internal standards) (Sigma). Supernatants (50 *μ*L) were then injected into an automated amino acid analyzer (JEOL Aminotac 500) and eluted with lithium citrate buffer. Tryptophan, ornithine, and arginine were detected at 570 nm. Data acquisition and calculations were made using the JEOL Workstation software.

### 2.11. Statistical Analysis

Mann Whitney *U* test was used to compare data between T cells alone, TIDC, and spDC. Results were considered statistically significant when *p* < 0.05. Data are expressed by the mean ± standard error of the mean (SEM). Analyses were performed with GraphPad Prism.

## 3. Results

### 3.1. CD11c^+^ Cells Infiltrating 4T1 and LLC Tumors Exhibit a Semimature DC Phenotype

**Figure 1:**
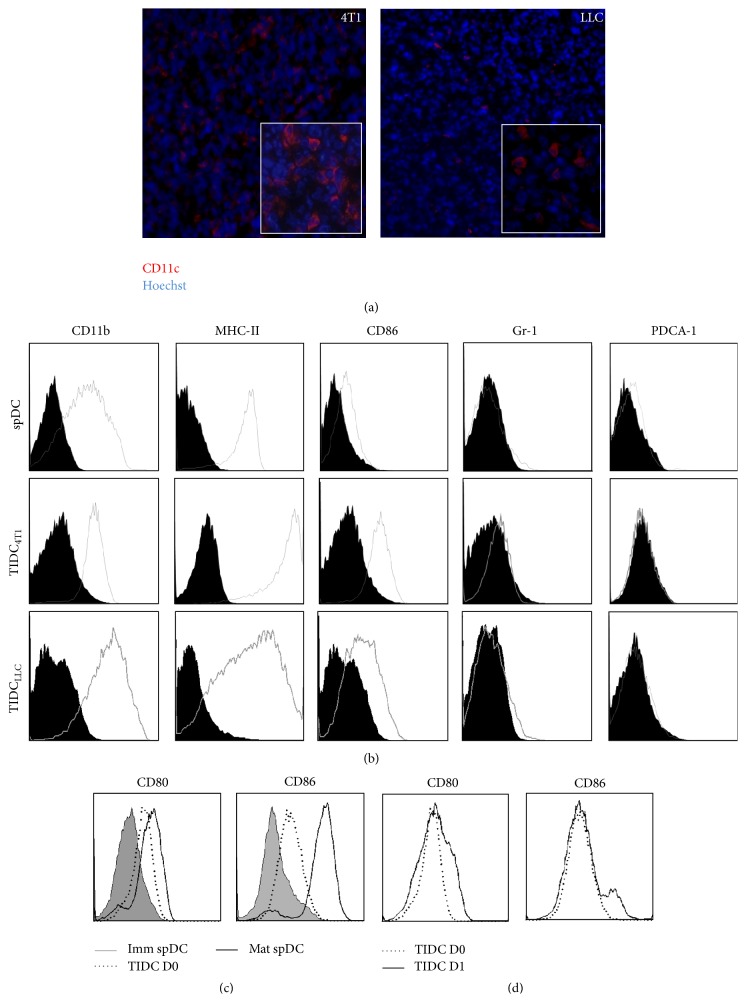
Murine 4T1 tumors are infiltrated with CD11c^+^CD86^+^MHC-II^+^ dendritic cells. (a) Frozen 4T1 tumor sections were stained with anti-CD11c antibodies and analyzed by inverted fluorescent microscopy. Tumor-infiltrating CD11c^+^ DC are shown in red (4T1* left panel* and LLC* right panel*). (b) CD11c^+^ cells isolated by magnetic cell sorting were further analyzed by cytometry for the expression of CD11b, MHC-II, CD86, Gr-1, and PDCA-1. (c, d) The expression of CD80 and CD86 by spDC or TIDC isolated from 4T1 tumor was evaluated immediately after isolation (D0) and after an overnight culture (D1). Dot plots quadrants were defined using isotype controls and the values are the percent of live cells in each quadrant. Results are representative of four independent experiments.

Differences in TIDC phenotype have been reported. Some studies have described an altered “immature” phenotype for DC infiltrating tumor tissues, while other reports have indicated that TIDC exhibit a mature phenotype [6, 7, 19]. The observed discrepancies may partly be explained by the type and stage of cancer. In our experiments, the murine mammary tumor 4T1 and the lung tumor LLC beds contain CD11c^+^ DC visualized by immunofluorescence or detected by flow cytometry (Figures 1(a) and 1(b)). These CD11c^+^ TIDC purified by magnetic cell sorting did not express Gr-1, a marker of myeloid-derived suppressor cells (MDSC) (Figure 1(b), Supplemental Figure  1 in Supplementary Material available online at http://dx.doi.org/10.1155/2015/891236) or PDCA-1, a marker of plasmacytoid DC (pDC) (Figure 1(b)). These data indicate that CD11c^+^ TIDC represent a population of cells phenotypically distinct from MDSC or pDC. Furthermore, TIDC expressed higher level of MHC Class II and of costimulatory molecules CD86 than CD11c^+^ spDC from naïve tumor-free mice (Figure 1(b)). We next compared the phenotype of freshly isolated TIDC to that of immature spDC (Imm spDC) that spontaneously matured over a period of 24 hrs* ex vivo*. Our data showed that TIDC expressed an intermediate level of costimulatory molecules compared to mature spDC (mat spDC, Figure 1(c)). The overnight* in vitro* culture of TIDC in complete medium which usually induces DC maturation did not lead to increased expression of CD80 and CD86 (Figure 1(d)). Collectively, these results indicate that CD11c^+^ TIDC exhibit a semimature phenotype and appeared to be blocked at this stage.

### 3.2. TIDC Isolated from 4T1 or LLC Tumors Suppress the Proliferation and Activation of T Lymphocytes

**Figure 2:**
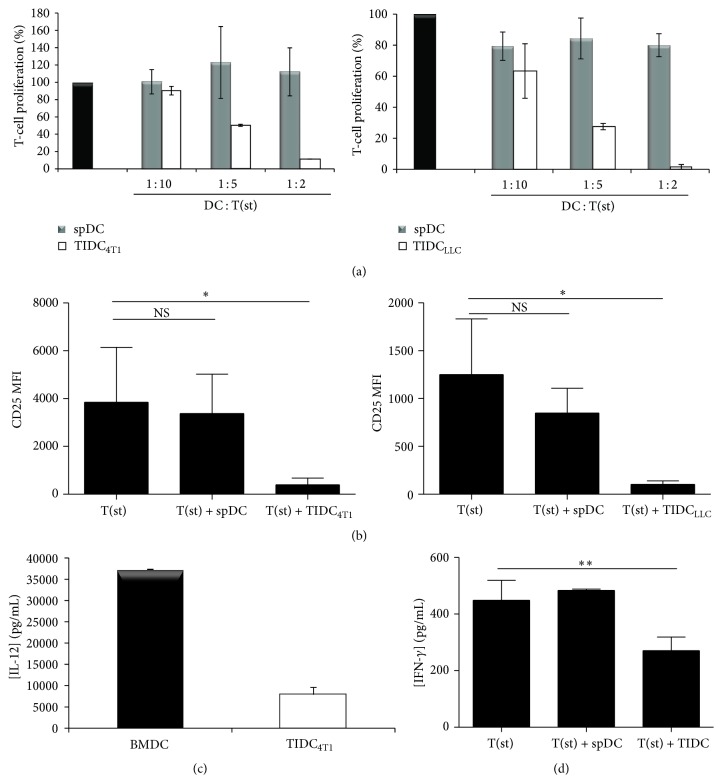
TIDC are immunosuppressive. (a) Autologous T-cell proliferation was measured after 5 days of culture with anti-CD3/anti-CD28-coated beads (T(st)) in the presence of spDC or TIDC isolated from 4T1 or LLC tumors at the indicated ratios. Data are representative of 5 independent experiments. (b) CD25 expression by T cells was measured after 5 days of culture with TIDC or spDC. Data are representative of 5 independent experiments. (c) IL-12p70 concentration was quantified by ELISA in TIDC isolated from 4T1 or BMDC 24 hr culture supernatant. (d) IFN-*γ* concentration was quantified in the supernatant of stimulated T cells cultured alone or in the presence of spDC or TIDC for 5 days.

Functionally competent mature DC are characterized by their capacity to induce T-cell proliferation and to produce high level of proinflammatory cytokines such as IL-12. The results depicted in Figure 2(a) indicate that CD11c^+^ TIDC isolated from 4T1 or LLC tumors were not capable of inducing allogeneic T lymphocyte proliferation. In fact, these TIDC inhibited, in a dose-dependent manner, the proliferation (Figure 2(a)) and activation (Figure 2(b)) of T lymphocytes induced* in vitro* with anti-CD3/anti-CD28-conjugated microbeads. These immunosuppressive properties were associated with a decrease in IL-12 production by the TIDC (Figure 2(c)). Consistent with these results, TIDC significantly impaired T-cell production of IFN-*γ* (Figure 2(d)). Altogether these data indicate that the 4T1 and LLC tumor microenvironment promotes the accumulation of CD11c^+^ DC with immunosuppressive activity. Since optimal inhibition of T lymphocyte activation and proliferation was observed at a TIDC: T-cell ratio of 1 : 2 (Figure 2(a)), subsequent experiments were performed using this ratio.

### 3.3. The Modulation of T-Cell Responses by CD11c^+^ TIDC Involves the Ectoenzyme CD39 Pathways

IL-10, iNOS, IDO, or arginase-1 has been reported as contributors of regulatory DC suppressive function. iNOS expression was substantially reduced in TIDC compared to bone marrow-derived DC (BMDC) (Supplemental Figure  2A, top). However, nitrites (byproducts of NO) were not detectable in TIDC culture medium (data not shown) and the iNOS inhibitor NMMA did not affect TIDC-mediated inhibition of T-cell proliferation (Supplemental Figure  2B). Similarly, blocking anti-IL10R antibodies did not prevent TIDC-mediated suppression of T-cell proliferation (data not shown). Although detectable IDO expression was observed in TIDC compared to control spDC (Supplemental Figure  2A, low), the IDO inhibitor 1-MT did not impair the suppressive function of these cells (Supplemental Figure  2B). Consistently, the concentration of tryptophan in the culture supernatant was similar whether T cells were cultured alone, or with spDC or TIDC, strongly suggesting that IDO was not activated (Supplemental Figure  2C, left). These results therefore indicate that IL-10, IDO, and iNOS are unlikely to play a significant role in the immunosuppressive function of TIDC.

**Figure 3:**
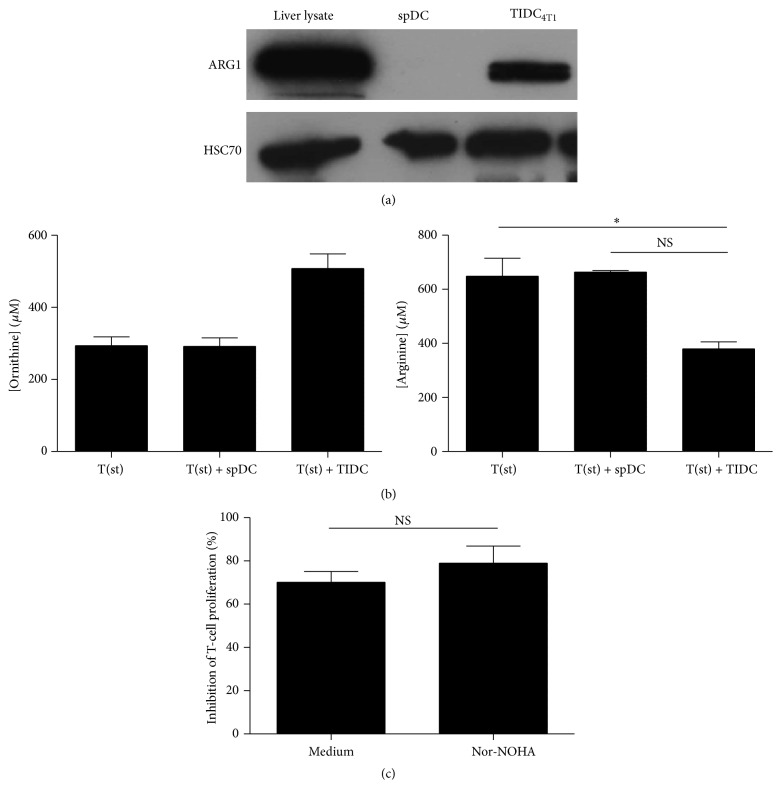
Immunosuppressive functions of TIDC depend on arginase-1 activity. (a) Expression of arginase-1 was evaluated in 4T1 tumor derived TIDC and in spDC by WB. Lysate of total liver was used as a positive control. (b) CD3/CD28-stimulated T cells were cultured alone, with TIDC or spDC. After 5 days, supernatants were collected and concentration of ornithine and arginine was quantified by HPLC. Columns represent the mean of the concentration and error bars the SEM. (c) Total T cells were stained using CellTrace Violet Cell Proliferation Kit, stimulated with CD3/CD28 beads and cultured for 5 days with TIDC or spDC in presence or absence of nor-NOHA, arginase-1 inhibitor. The proliferation was defined by flow cytometry and analyzed by ModFit software. The percentage of inhibition for each condition was calculated based on the proliferation index of stimulated T cells (representative of 5 experiments).

Previous reports have indicated that arginase-1 was involved in TIDC suppressive function [6]. The data depicted in Figure 3(a) indicate that the expression of this enzyme by TIDC was enhanced compared to control spDC. Consistent with this result the production of ornithine (generated by arginase-1) was increased in T-cell and TIDC coculture supernatants (Figure 3(b), left), which was associated with a decreased concentration of arginine (Figure 3(b), right). Intriguingly, arginase-1 inhibitor, nor-NOHA, is not sufficient to induce a significant decrease of the TIDC-mediated suppression of T-cell proliferation (Figure 3(c)).

**Figure 4:**
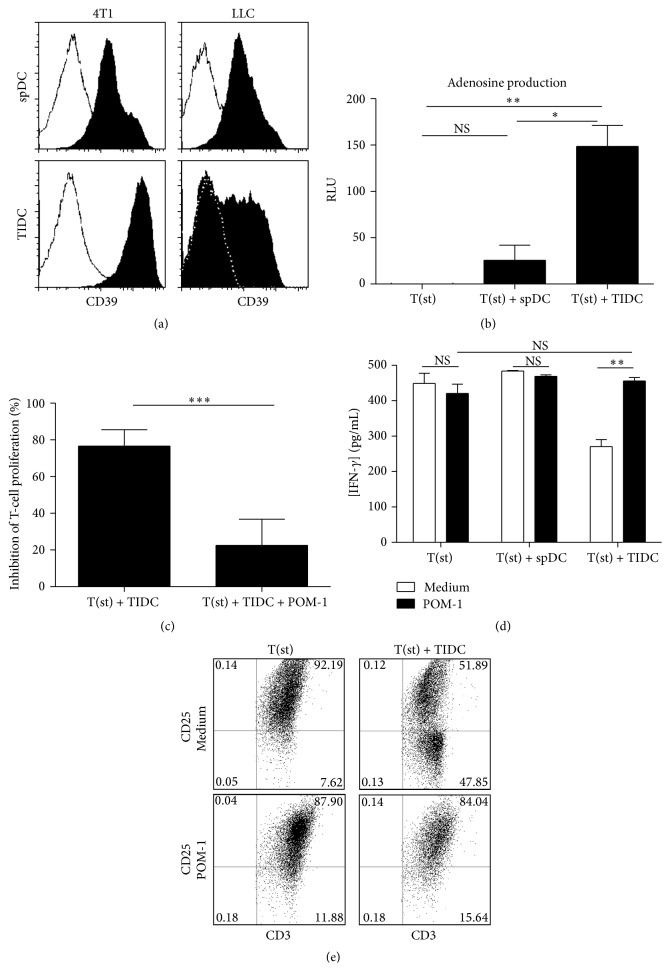
TIDC inhibit T-cell response through the ectoenzyme CD39. (a) Purified spDC and TIDC (derived from 4T1 and LLC tumors) were stained for CD39. The white histograms represent the isotype controls (representative of 5 independent experiments). (b) CD3/CD28-stimulated T cells were cultured alone (T(st)), with TIDC (T(st) + TIDC), or with spDC (T(st) + spDC). After 48 hours, supernatants were collected and concentration of adenosine was determined by chemiluminescence assay kits (representative of 3 experiments). (c) Total T cells were labeled with CellTrace Violet, stimulated with CD3/CD28 beads and cultured for 5 days with TIDC in presence or absence of POM-1, the CD39 inhibitor. The proliferation index was measured and the percentage of inhibition for each condition was calculated based on the proliferation index of stimulated T cells. (d) IFN-*γ* concentration was quantified in the supernatant of the previous cocultures. (e) The T cells exposed to TIDC or spDC were stained for CD25 and CD3 to evaluate T-cell activation.

The ectoenzyme CD39 is responsible for the production of adenosine, a molecule described for its ability to suppress T-cell proliferation and cytokine production [30, 31]. The ectoenzyme CD39 has also been reported for its role in Treg suppressive activity [31]. TIDC from tumor-bearing mice or spDC from naïve mice both expressed CD39, with however a much higher level of expression of this enzyme by TIDC (Figure 4(a)). Consistent with these data, adenosine levels were higher in the supernatant of activated T cells cultured with TIDC (Figure 4(b)). Furthermore, inhibition of CD39 activity using POM-1, a pharmacologic NTPDase inhibitor, partially decreased TIDC suppressive activity (Figure 4(c)) and adenosine production (data not shown). Moreover, POM-1-treated TIDC failed at inhibiting IFN-*γ* production (Figure 4(d)) and CD25 expression by T lymphocytes (Figure 4(e)). Taking together, these results therefore strongly suggest that TIDC-mediated suppression of T-cell proliferation involves a complex of mechanisms including CD39 pathways.

## 4. Discussion

Tumor-induced immunosuppression represents a major impediment to successful cancer immunotherapeutic strategies leading to T lymphocyte activation. During the last decades, extensive research has focused on identifying populations of immunosuppressive cells induced and recruited by developing tumors and on deciphering the associated immunosuppressive mechanisms [32, 33]. In this context, TIDC have been highlighted as essential constituents of these immunoinhibitory networks [34].

Consistent with previous studies conducted with different cancer types [6, 7, 14, 35, 36], CD11c^+^ TIDC isolated from 4T1- or LLC-tumor bearing mice exhibit an altered phenotype characterized by intermediary expression of costimulatory (CD80, CD86, and CD40) and MHC II molecules, produce low amounts of the proinflammatory cytokine IL-12, and are poor inducers of T lymphocyte proliferation. Importantly, these cells can not further mature spontaneously* in vitro*, suggesting a blockade at this stage of differentiation [35, 37]. Whether recently reported approaches to activate TIDC (for instance, the synergistic stimulation of CD40 and TLR3 [38] or miRNA mimetics [39]) may promote the maturation of TIDC isolated from 4T1 or LLC has however not been evaluated in our current study.

TIDC isolated from 4T1 or LLC tumors efficiently suppress CD4^+^ and CD8^+^ T cells, therefore clearly demonstrating their immunosuppressive properties. Identifying the mechanisms employed by TIDC to impair antitumor immune response is essential for the design of therapeutic strategies to overcome the tumor-promoting influence of these cells. Different mechanisms underlying TIDC immunosuppressive activity have been reported [6, 10, 25, 40–42]. Recently, Norian et al. have demonstrated that the immunoinhibitory function of DC isolated from murine mammary tumors depends on arginase-1 [6]. In addition, different immunosuppressive cells such as MDSC or tumor-associated fibroblasts have been reported to impair T lymphocyte function through increased L-arginine catabolism [10, 37, 43]. Since 4T1 tumors are characterized by strong expression of IL-6, which has been involved in the regulation of arginase-1 expression [44, 45], the possible role of this enzyme was evaluated. CD11c^+^ TIDC from 4T1 tumors highly express arginase-1. Consistently, a decrease in L-arginine associated with an increase in ornithine concentration was detected in the supernatant of stimulated T cells cultured with TIDC compared to stimulated T cells cultured with spDC. These observations suggest that arginine depletion may contribute to the TIDC-induced T-cell inhibition. However, despite a 40% decrease in the arginine level, the arginine concentration is still superior to 50 *μ*M, which is the higher concentration inducing T-cell immunosuppression [46]. Moreover, the use of arginase-1 inhibitor, nor-NOHA, did not restore T-cell proliferation. So the implication of arginase-1 in the immunosuppressive function of TIDC remains unclear.

Further investigation of the mechanisms responsible for TIDC suppressive function highlighted for the first time the role of the ectonucleotidase CD39. We indeed observed that the specific CD39 inhibitor POM-1 decreases the capability of CD11^+^ TIDC to suppress T-cell proliferation and IFN-*γ* secretion. Interestingly, several immune cells which are not always immunoinhibitor such as B and T lymphocytes, monocytes, Langerhans cells, and natural killer cells have been reported to express CD39 [47, 48]. Similarly, nonsuppressive memory human or murine T-cell populations can also express CD39 [49, 50]. Recently, several groups have independently reported on the expression of CD39 by Treg, cancer exosomes, tumor cells, and multipotent mesenchymental stromal cells [30, 51, 52]. To exert its function, the ectonucleotidase CD39 cooperates with other enzymes, the best known of which is CD73. The tandem of CD39/CD73 is responsible of the hydrolysis of extracellular ATP and ADP to AMP (by CD39) and the conversion of AMP to adenosine (by CD73). Adenosine, by binding to A2A receptors, leads to the accumulation of intracellular cAMP, thereby blocking the TCR signaling and consequently the T-cell proliferation [53–55]. The strong production of adenosine shown in our results demonstrates that the CD39-adenosine pathway is involved in the T-cell proliferation inhibition by TIDC.

Our results therefore indicate that T lymphocyte inhibition is mediated by CD11c^+^ TIDC via several suppressive mechanisms among which CD39 plays an important role. These immune regulatory mechanisms may therefore represent important new targets of therapeutic strategies aimed at reversing TIDC negative impact in cancer.

## Supplementary Material

Supplementary Figure 1: the expression of the following markers (CD11b, MHC-II, GR-1 and CD11c) by TIDC and MDSC were studied by flow cytometry and the data are shown in this figure.
Supplementary Figure 2: The involvement of iNOS and IDO enzymes in the immunosuppressive function of TIDC was assessed and the data are shown in this figure.
