# Supplementary material for: T Lymphocyte Inhibition by Tumor-Infiltrating Dendritic Cells Involves Ectonucleotidase CD39 but Not Arginase-1
Source: Biomed Res Int. 2015 Sep 30;2015:891236. doi: 10.1155/2015/891236 (PMC4605267; doi:10.1155/2015/891236)
Supplement: Supplementary file 1 — Supplementary Figure 1: the expression of the following markers (CD11b, MHC-II, GR-1 and CD11c) by TIDC and MDSC were studied by flow cytometry and the data are shown in this figure. Supplementary Figure 2: The involvement of iNOS and IDO enzymes in the immunosuppressive function of TIDC was assessed and the data are shown in this figure. [file 891236.f1.zip › Trad at al Sup Figure 2.pptx]

## Slide 1
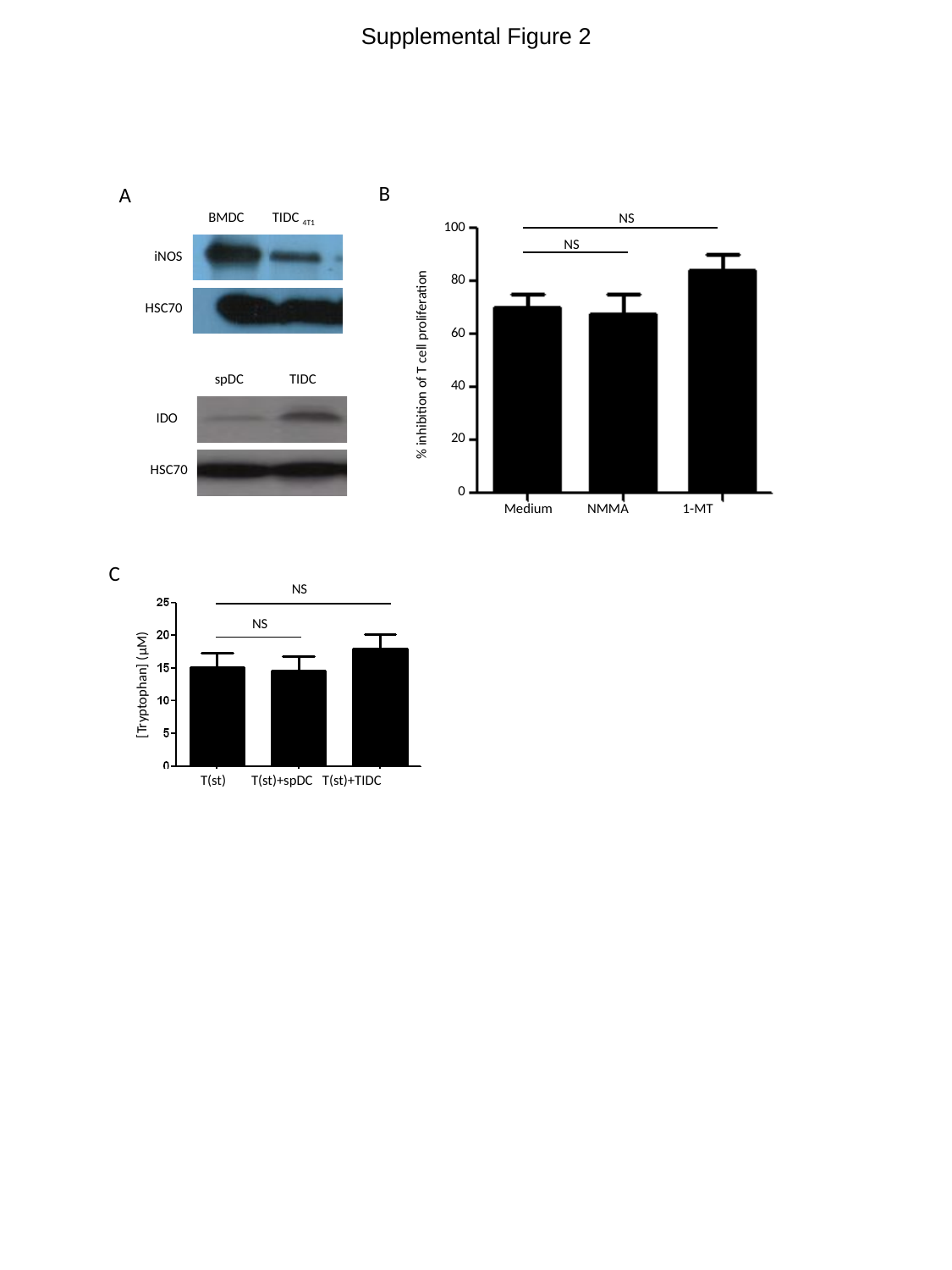

Supplemental Figure 2
B
NS
100
NS
80
60
% inhibition of T cell proliferation
40
20
0
 Medium NMMA 1-MT
A
BMDC
TIDC 4T1
iNOS
HSC70
spDC
TIDC
HSC70
IDO
C
NS
NS
[Tryptophan] (µM)
 T(st) T(st)+spDC T(st)+TIDC
