# Supplementary figures and images for: T Lymphocyte Inhibition by Tumor-Infiltrating Dendritic Cells Involves Ectonucleotidase CD39 but Not Arginase-1
Source: Biomed Res Int. 2015 Sep 30;2015:891236. doi: 10.1155/2015/891236 (PMC4605267; doi:10.1155/2015/891236)

## Slide 1
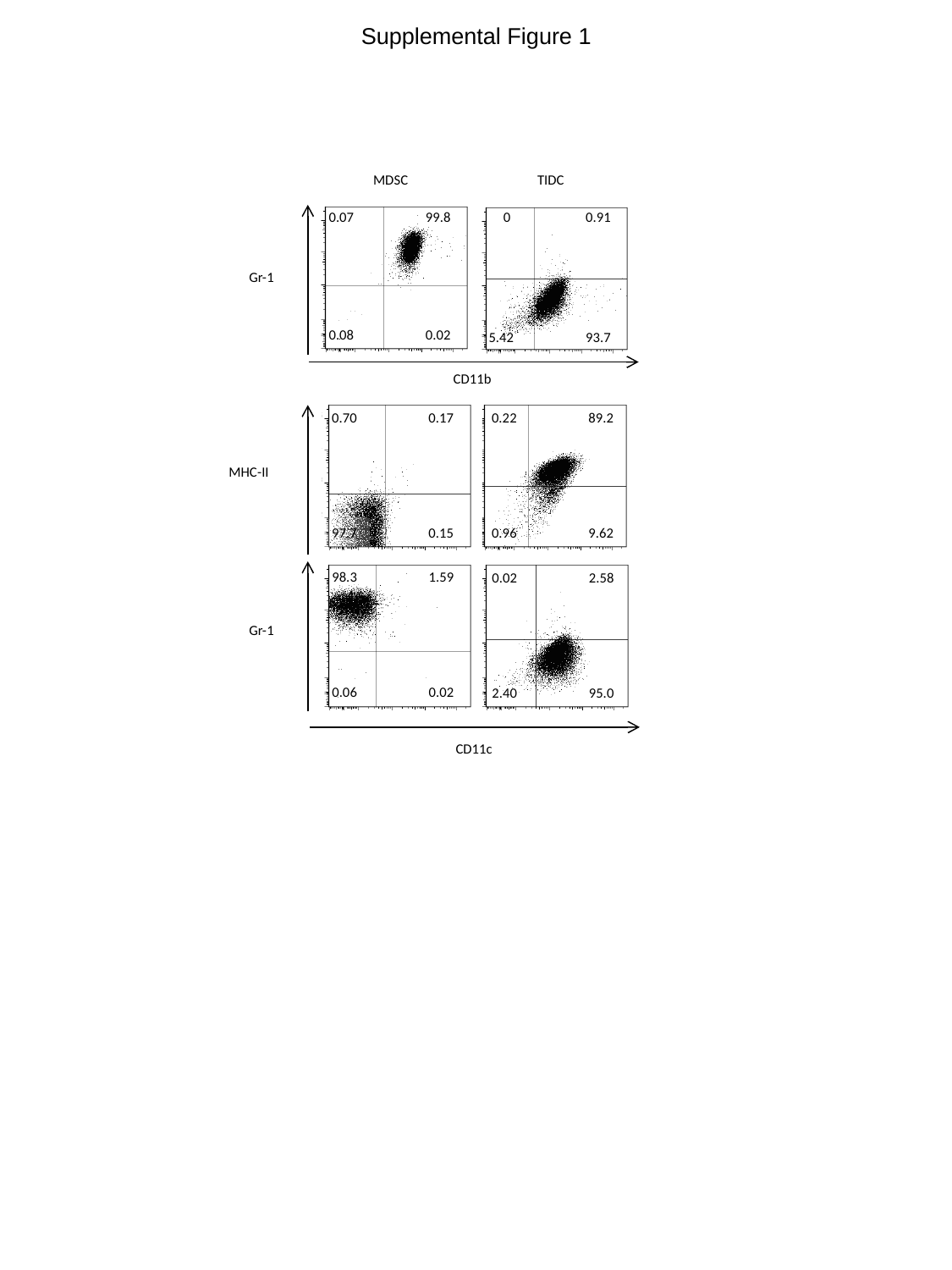

Supplemental Figure 1
MDSC
TIDC
0.07
99.8
0.08
0.02
0
0.91
93.7
5.42
Gr-1
CD11b
0.70
0.17
97.7
0.15
0.22
89.2
0.96
9.62
MHC-II
98.3
1.59
0.02
0.06
0.02
2.58
2.40
95.0
Gr-1
CD11c

Supplement: Supplementary file 1 — Supplementary Figure 1: the expression of the following markers (CD11b, MHC-II, GR-1 and CD11c) by TIDC and MDSC were studied by flow cytometry and the data are shown in this figure. Supplementary Figure 2: The involvement of iNOS and IDO enzymes in the immunosuppressive function of TIDC was assessed and the data are shown in this figure. [file 891236.f1.zip › Trad at al Sup Figure 1.pptx]
